# A-803467, a tetrodotoxin-resistant sodium channel blocker, modulates ABCG2-mediated MDR *in vitro* and *in vivo*

**DOI:** 10.18632/oncotarget.5747

**Published:** 2015-10-22

**Authors:** Nagaraju Anreddy, Atish Patel, Yun-Kai Zhang, Yi-Jun Wang, Suneet Shukla, Rishil J. Kathawala, Priyank Kumar, Pranav Gupta, Suresh V. Ambudkar, John N. D. Wurpel, Zhe-Sheng Chen, Huiqin Guo

**Affiliations:** ^1^ Department of Pharmaceutical Sciences, College of Pharmacy and Health Sciences, St. John's University, Queens, NY 11439, USA; ^2^ Laboratory of Cell Biology, Center for Cancer Research, National Cancer Institute, National Institutes of Health, Bethesda, MD 20892, USA; ^3^ Department of Thoracic Surgery, Peking Union Medical College Hospital, Beijing 100730, P.R. China

**Keywords:** multidrug resistance, ABCG2, ABC transporters, non-small cell lung cancer

## Abstract

ATP-binding cassette subfamily G member 2 (ABCG2) is a member of the ABC transporter superfamily proteins, which has been implicated in the development of multidrug resistance (MDR) in cancer, apart from its physiological role to remove toxic substances out of the cells. The diverse range of substrates of ABCG2 includes many antineoplastic agents such as topotecan, doxorubicin and mitoxantrone. ABCG2 expression has been reported to be significantly increased in some solid tumors and hematologic malignancies, correlated to poor clinical outcomes. In addition, ABCG2 expression is a distinguishing feature of cancer stem cells, whereby this membrane transporter facilitates resistance to the chemotherapeutic drugs. To enhance the chemosensitivity of cancer cells, attention has been focused on MDR modulators. In this study, we investigated the effect of a tetrodotoxin-resistant sodium channel blocker, A-803467 on ABCG2-overexpressing drug selected and transfected cell lines. We found that at non-toxic concentrations, A-803467 could significantly increase the cellular sensitivity to ABCG2 substrates in drug-resistant cells overexpressing either wild-type or mutant ABCG2. Mechanistic studies demonstrated that A-803467 (7.5 μM) significantly increased the intracellular accumulation of [^3^H]-mitoxantrone by inhibiting the transport activity of ABCG2, without altering its expression levels. In addition, A-803467 stimulated the ATPase activity in membranes overexpressed with ABCG2. In a murine model system, combination treatment of A-803467 (35 mg/kg) and topotecan (3 mg/kg) significantly inhibited the tumor growth in mice xenografted with ABCG2-overexpressing cancer cells. Our findings indicate that a combination of A-803467 and ABCG2 substrates may potentially be a novel therapeutic treatment in ABCG2-positive drug resistant cancers.

## INTRODUCTION

Multidrug resistance (MDR) is defined as the resistance of cancer cells to antineoplastic agents that have distinct structures and mechanisms of action [1]. Particularly, cancer cells are said to have an MDR phenotype when the cells show cross-resistance to drugs that are structurally and functionally unrelated to the original drug which the cells are resistant [2]. Once MDR is acquired, the efficacy of chemotherapeutic drugs decreases. MDR is the most significant hindrance to successful chemotherapy, and is a main cause of cancer metastasis and relapse [3]. Chemotherapy resistance can be primary, which is initially determined by a refractory response to the pharmacotherapy, or secondary/acquired, which is developed during treatment progression [3].

The potential mechanisms of MDR include pharmacokinetic alterations, tumor micro-environmental changes, or cancer cell-specific factors that occur at different levels due to cellular alterations, which include increased drug efflux or decreased drug uptake, drug inactivation, drug target modification or apoptosis evasion [4]. The key effectors of drug resistance are multidrug transporters which can be elevated or hyper-activated during the genesis of drug resistant cancers. The ATP-binding cassette (ABC) multidrug transporters such as ABCB1 (MDR1/P-glycoprotein), ABCC1 (MRP1) and ABCG2 (BCRP/MXR) are considered to be accountable for the majority of drug efflux in human cancers [5]. The ABCG2 transporter is a 72-kDa half transporter, which was identified from a doxorubicin-selected MCF-7 human breast cancer cell line [6], human placenta [7], and a colon cancer cell line (S1-M1–80) [8]. ABCG2 is specifically localized at the apical surface of enterocytes, the luminal surface of liver canaliculi, the luminal surface of the proximal convoluted tubule of the kidneys, the blood–brain barrier (BBB), blood–testis barrier (BTB), blood–placental and blood–retinal barriers. Because of its localization on the secretory surface of the major organs involved in drug transport, ABCG2 alters the ADME (absorption, distribution, metabolism and elimination) of its substrate drugs. ABCG2 can transport large, hydrophobic, positively and negatively charged molecules, including cytotoxic compounds such as mitoxantrone (MX), topotecan, flavopiridol and methotrexate [9]. Although the contribution of ABCG2 in clinical MDR has not been completely investigated, some studies have described the association between ABCG2 expression and poor chemotherapeutic response. Increased ABCG2 gene expression has also been related to poor response to chemotherapy in childhood acute myeloid leukemia (AML) and relapsed AML [10, 11]. ABCG2 expression has also been reported in leukemia, especially in pediatric AML [12]. In addition, increased *ABCG2* mRNA has been reported in irinotecan treated hepatic metastases compared to irinotecan-naive metastases [13]. ABCG2 expression has been reported in various solid tumors, such as those present in the digestive tract, endometrium and melanoma [14].

Recently, ABCG2 has been recognized as a molecular marker for the side population (SP) cells, these are putative cancer stem cell CSC population. SP cells are identified using dual wavelength flow cytometry combined with Hoechst 33342 dye efflux [15]. For human Non-Small Cell Lung Cancer (NSCLC) cell lines, excluding 0.03 - 6.1% of the tumor cells which were SP cells [16], the presence of a Hoechst dye 33342 showed elevated expression of ABCG2, an increased tumorigenicity in mice resistant to various chemotherapeutic agents [17]. Moreover, Yoh et al. found that positive immunostaining for ABCG2 appears to be a predictor of shorter survival in patients with advanced NSCLC [18]. Until now, several ABCG2 inhibitors with diverse chemical structures have been found or developed, but none of them have been tested clinically due to concerns of toxicity, safety or the pharmacokinetic uncertainty of the compounds [19].

A-803467 is a potent and selective Na_v_1.8 sodium channel blocker, which has shown significant anti-nociception in animal models of neuropathic and inflammatory pain [20]. Previously, ion channel inhibitors such as verapamil and quinidine have shown to reverse ABC transporter mediated MDR [21]. We, and others, have further reported several natural drugs, marine drugs, semi-synthetic and synthetic compounds which could reverse ABCG2-mediated MDR [22–25]. Therefore, here we determine A-803467 as a therapeutic compound to enhance the chemosensitivity of conventional anticancer drugs through interaction with the ABCG2 transporter.

## RESULTS

### A-803467 significantly increases the cytotoxicity of anticancer drugs which are substrates of ABCG2, but not of ABCB1 and ABCC10

Cytotoxicity of A-803467 treatment alone on ABCG2-overexpressing cell lines was investigated and found to be nontoxic with IC_50_ values greater than 10 μM (Supplementary Figure S1). Accordingly, reversal concentrations of 2.5 and 7.5 μM, at which no significant cytotoxicity was detected for A-803467 alone, were chosen for further experiments. HEK293 cells transfected with wild-type (HEK293/R482) and mutant (HEK293/R482G and HEK293/R482T) ABCG2 (Supplementary Figure S2) showed significant resistance to MX and topotecan compared to HEK293/pcDNA3.1 (Table 1). The test compound A-803467 at 7.5 μM significantly increased the cytotoxicity of MX and topotecan in ABCG2-transfected cell lines (Table 1). In addition, the reversal effect of A-803467 on ABCG2-mediated MDR was comparable to the effect produced by 5 μM of FTC, a known ABCG2 inhibitor. However, A-803467 did not sensitize ABCG2-transfected cells to cisplatin, a non-substrate of ABCG2 (Table 1). Furthermore, the reversal effect of A-803467 was also analyzed in parental H460, and drug selected ABCG2 overexpressing H460/MX20 cells. We found similar results where A-803467 significantly increased the cytotoxicity of MX and topotecan in ABCG2 overexpressing H460/MX20 cells (Table 2). However, A-803467 did not sensitize the parental H460 cells to MX and topotecan (Table 2). Independently, we also analyzed the effect of A-803467 on ABCB1- and ABCC10-mediated MDR. We found that A-803467 did not affect the ABCB1- and ABCC10-mediated MDR in ABCB1 overexpressing HEK293/ABCB1 cells and ABCC10 overexpressing HEK293/ABCC10 cells, respectively (Table 3). Together these results indicate that A-803467 selectively and significantly reverses the ABCG2-mediated MDR.

**Table 1:** A-803467 enhances the cytotoxicity of mitoxantrone and topotecan in HEK293/pcDNA3.1 cells overexpressing the wild-type as well as mutant ABCG2

Treatments	IC_50_ ± SD (nM)							
	HEK293/pcDNA3.1	FR	HEK293/R482	FR	HEK293/R482G	FR	HEK293/R482T	FR
Mitoxantrone	24.8 ± 0.9	1.0	258.5 ± 12.8	10.4#	723.8 ± 12.5	29.1#	808.0 ± 38.2	32.4#
+A-803467 (2.5 μM)	21.5 ± 0.8	0.8	57.5 ± 0.9	2.3*	66.2 ± 1.2	2.6*	74.0 ± 18.9	3.0*
+A-803467 (7.5 μM)	20.4 ± 2.0	0.9	19.5 ± 0.2	0.8*	24.2 ± 1.5	0.9*	34.5 ± 16.7	1.3*
+FTC (5 μM)	21.5 ± 0.8	0.8	17.7 ± 0.1	0.7*	22.4 ± 1.2	0.9	36.5 ± 18.7	1.4*
Topotecan	10.2 ± 2.5	1.0	280.9 ± 30.6	27.5	224.2 ± 12.6	22.0	187.2 ± 19.6	18.4
+A-803467 (2.5 μM)	10.5 ± 3.6	0.9	182.3 ± 23.8	17.9	131.4 ± 21.6	12.9	137.7 ± 15.6	13.5
+A-803467 (7.5 μM)	9.4 ± 3.7	0.9	18.6 ± 4.6	1.8*	15.3 ± 2.8	1.5*	17.4 ± 3.8	1.7*
+FTC (5 μM)	9.8 ± 2.8	0.9	19.8 ± 2.5	1.9*	16.4 ± 2.4	1.6*	16.9 ± 1.4	1.6*
Cisplatin	2945.8 ± 55.9	1.0	2636.0 ± 94.1	0.9	2566.4 ± 88.2	0.8	2745.6 ± 141.8	0.9
+A-803467 (2.5 μM)	2486.7 ± 90.1	0.8	2486.5 ± 125.5	0.8	2478.8 ± 70.6	0.8	2399.4 ± 106.4	0.8
+A-803467 (7.5 μM)	2507.6 ± 186.1	0.8	2377.7 ± 125.3	0.8	2378.2 ± 55.5	0.8	2377.7 ± 125.3	0.8
+FTC (5 μM)	2641.4 ± 84.2	0.8	2396.2 ± 127.02	0.8	2367.5 ± 27.6	0.9	2347.7 ± 43.5	0.8

Data represents the mean IC_50_ values for each cell line ± SD obtained from three independent sets of experiments. Statistical analysis was performed by One-way ANOVA followed by Newman-Keuls Multiple Comparison Test. Statistical significance was set at *P* < 0.05.

* *P* < 0.05 versus the control group.

# *P* < 0.05 versus the control of HEK293/pcDNA3.1 group. The fold resistance (FR) was determined by dividing the IC_50_ value of anticancer drug for HEK293/pcDNA3.1, HEK293/R482, HEK293/R482G and HEK293/R482T, in the absence or presence of reversal agents, by the IC_50_ value of respective anticancer drug for HEK293/pcDNA3.1 in the absence of reversal agent. FTC was used as a positive control of ABCG2 inhibitor

**Table 2:** A-803467 enhances the cytotoxicity of ABCG2 substrate anticancer drugs in H460/MX20 cells overexpressing ABCG2

Treatments	IC_50_ ± SD (nM)				
	H460	FR	H460/MX20	FR
**Mitoxantrone**	58.5 ± 4.2	1.0	7956.0 ± 198.2	135.9#
+A-803467 (2.5 μM)	57.9 ± 1.8	0.9	773.0 ± 18.9	13.2*
+A-803467 (7.5 μM)	55.2 ± 1.3	0.9	345.5 ± 16.7	5.9*
+FTC (5 μM)	55.3 ± 2.3	0.9	363.5 ± 18.7	6.2*
**Topotecan**	23.4 ± 0.8	1.0	1258.8 ± 47.4	53.3#
+A-803467 (2.5 μM)	20.9 ± 1.2	0.8	445.8 ± 24.7	19.0*
+A-803467 (7.5 μM)	19.3 ± 0.8	0.8	94.4 ± 5.2	4.0*
+FTC (5 μM)	19.7 ± 0.4	0.8	112.5 ± 8.2	4.8*
**Cisplatin**	2225.5 ± 12.3	1.0	2147.8 ± 24.3	0.9
+A-803467 (2.5 μM)	2147 ± 24.3	0.9	2143.9 ± 42.2	0.9
+A-803467 (7.5 μM)	2209.1 ± 42.3	0.9	2170 ± 72.1	0.9
+FTC (5 μM)	2126.2 ± 50.9	0.9	2172.8 ± 49.9	0.9

Data represents the mean IC_50_ values for each cell line ± SD obtained from three independent sets of experiments. Statistical analysis was performed by One-way ANOVA followed by Newman-Keuls Multiple Comparison Test. Statistical significance was set at *P* < 0.05.

* *P* < 0.05 versus the control group.

# *P* < 0.05 versus the control of H460 group. The fold resistance (FR) was determined by dividing the IC_50_ value of the anticancer drug for H460 and H460/MX20, in the absence or presence of reversal agents, by the IC_50_ value of the respective anticancer drug for H460, in the absence of reversal agent. FTC was used as a positive control of ABCG2 inhibitor.

**Table 3:** A-803467 cannot enhance the cytotoxicity of ABCB1 and ABCC10 substrate anticancer agents in HEK293/PCDNA3.1 cells overexpressing ABCB1 and ABCC10

Treatments	IC_50_ ± SD (nM)					
	HEK293/pc DNA3.1	FR	HEK293/ABCB1	RF	HEK293/ABCC10	FR
**Paclitaxel**	8.3 ± 0.2	1.0	525.2 ± 20.1	63.2#	95.2 ± 6.1	11.4#
+A-803467 (7.5 μM)	7.9 ± 0.4	0.9	453 ± 18.9	54.5	77.4 ± 5.6	9.3
+Verapamil (5 μM)	8.2 ± 0.6	1.0	9.5 ± 1.5	1.0 *	−	−
+Cepharanthine (2.5 μM)	7.2 ± 0.3	0.8	−	−	12.3 ± 2.5	1.4*

Data represents the mean IC_50_ values for each cell line ± SD obtained from three independent sets of experiments. Statistical analysis was performed by One-way ANOVA followed by Newman-Keuls Multiple Comparison Test. Statistical significance was set at *P* < 0.05.

* *P* < 0.05 versus the control group.

# *P* < 0.05 versus the control of HEK293/pcDNA3.1 group.

The fold resistance (FR) was determined by dividing the IC_50_ value of the anticancer drug for HEK293/pcDNA3.1, HEK293/ABCB1 and HEK293/ABCC10, in the absence or presence of reversal agents, by the IC_50_ value of the respective anticancer drug for HEK293/pcDNA3.1 in the absence of reversal agent. Verapamil and cepharanthine were used as positive control inhibitors for ABCB1 and ABCC10 respectively.

### A-803467 significantly augments the intracellular accumulation of [^3^H]-MX in cells overexpressing ABCG2

**Figure 1:**
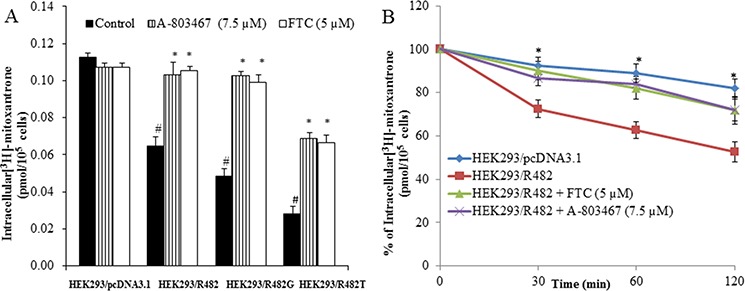
Effect of A-803467 on the accumulation and efflux of [3H]-MX in ABCG2-expressing cells **A.** A-803467 at 7.5 μM significantly increased intracellular accumulation of [^3^H]-MX in ABCG2-expressing cells HEK293/R482, HEK293/R482G and HEK293/R482T cells.*: *P* < 0.05 versus the control group. ^#^: *P* < 0.05 versus the control of HEK293/pc DNA 3.1 group. **B.** The efflux activity of ABCG2 was significantly inhibited by 7.5 μM of A-803467 at 0, 30, 60, and 120 min of treatment in HEK293/R482 cells. *: *P* < 0.05 versus the HEK293/R482 group.

To investigate the reversal mechanism, we studied the effect of A-803467 on the intracellular accumulation of [^3^H]-MX in ABCG2 overexpressing cells. HEK293/pcDNA3.1 and ABCG2-transfected cells were incubated with [^3^H]-MX, a known substrate of ABCG2, with or without A-803467 at different concentrations for 2 h. A-803467 at 7.5 μM significantly enhanced the intracellular [^3^H]-MX accumulation in ABCG2-transfected cells. However, A-803467 did not significantly impact the intracellular accumulation in HEK293/pcDNA3.1 cells (Fig. 1A). These results suggest that the increased intracellular levels of [^3^H]-MX in ABCG2 overexpressing cells is due to the inhibitory effect of A-803467 on the drug efflux function of the ABCG2 transporter.

### A-803467 decreases the efflux of [^3^H]-MX in cells overexpressing ABCG2

We accomplished a time course [^3^H]-MX efflux, with or without A-803467, in ABCG2-transfected cells to determine if the increase in intracellular [^3^H]-MX accumulation caused by A-803467 was due to inhibition of [^3^H]-MX efflux. We observed the efflux rate of [^3^H]-MX was significantly higher in ABCG2-transfected cells as compared with HEK293/pcDNA3.1 cells. A-803467 at 7.5 μM significantly blocked the intracellular [^3^H]-MX efflux at different time periods (0, 30, 60 and 120 min) from ABCG2-transfected cells, but not in the parental HEK293/pcDNA3.1 cells. In the absence of A-803467, the accumulation of [3H]-MX in HEK293/R482 cells at 30, 60 and 120 min were 72.5%, 62.7% and 52.4%, respectively. When HEK293/R482 cells were incubated with A-803467, the percentages of [^3^H]-MX at 30, 60 and 120 min were increased to 86.5%, 84.3% and 72.0%, respectively (Fig. 1B).

### A-803467 does not alter the total expression or translocation of ABCG2

**Figure 2:**
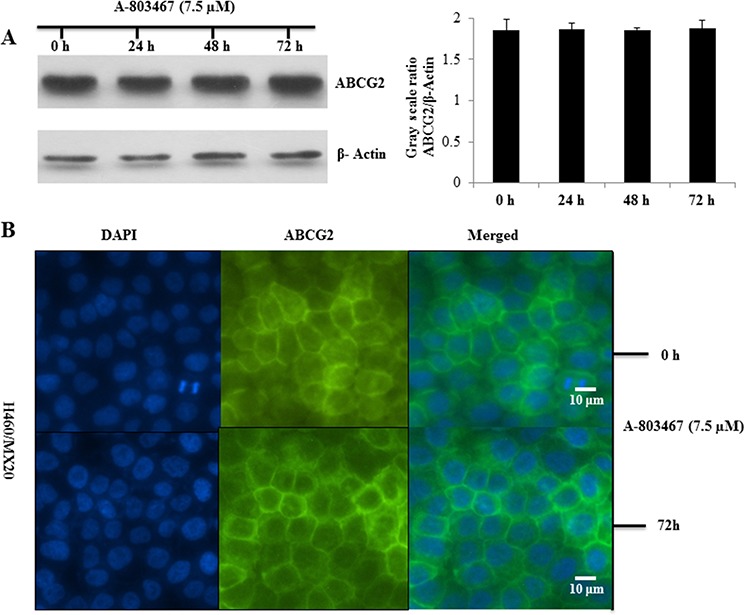
Effect of A-803467 on ABCG2 expression and the subcellular localization of ABCG2 **A.** Effect of A-803467 at 7.5 μM on the expression level of ABCG2 in H460/MX20 cell line. The protein levels of ABCG2 were normalized to those of actin. Values show the mean ± SD of 3 assays. **B.** Effect of A-803467 treatment on the subcellular localization of ABCG2 in H460/MX20 cell. ABCG2 staining is shown in green. DAPI (blue) counterstains the nuclei.

To analyze the effect of A-803467 on the ABCG2 expression, we incubated H460/MX20 cells with A-803467 (7.5 μM) for 24, 48, and 72 h and found that there was no significant change in the expression level of ABCG2 upon A-803467 treatment (Fig. 2A). To analyze if A-803467 causes a translocation of ABCG2 from the plasma membrane to the cytoplasm, contributing to a reduction of functional ABCG2, we performed an immunofluorescence analysis with H460 and ABCG2 overexpressing H460/MX20 cells. The results showed that the membrane expression and location of ABCG2, in H460/MX20 cells, was not altered after treatment with A-803467 (7.5 μM) for 72 h (Fig. 2B).

### A-803467 stimulates the ATPase activity of ABCG2

**Figure 3:**
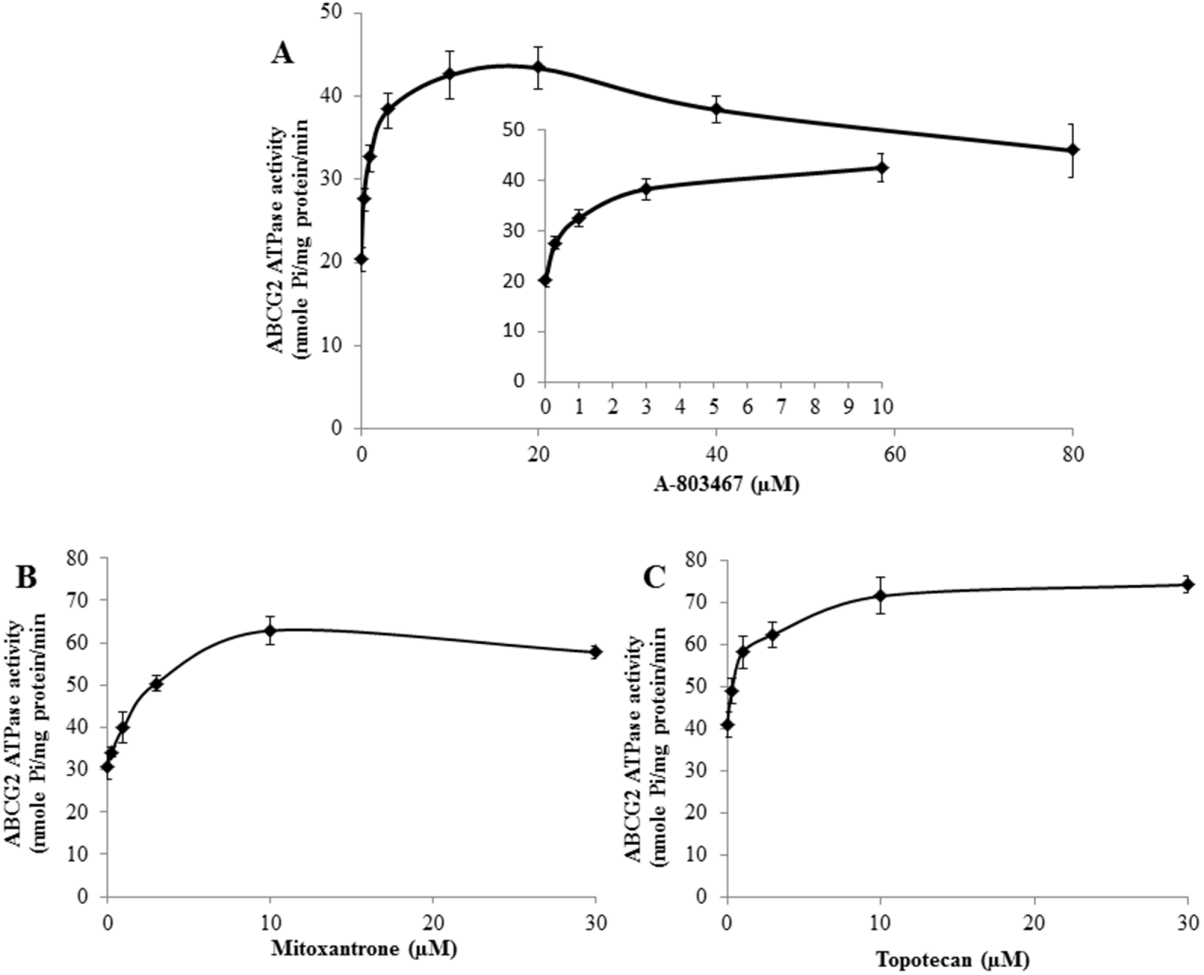
Effect of A-803467, MX and topotecan on the ATPase activity of ABCG2 **A.** Vanadate-sensitive ATPase activity of ABCG2 in membrane vesicles was determined with different concentrations of A-803467, as described in materials and methods. A-803467 showed a concentration dependent increase in ATPase activity. **B.** Effect of MX on the ATPase activity of ABCG2. **C.** Effect of topotecan on the ATPase activity of ABCG2. All values are the mean ± SD of 3 assays.

Several reversal agents have been reported as an inhibitor and/or substrate of ABC transporters [33–36]. To determine interaction of A-803467 with ABCG2 ATPase, we performed an ATPase assay using membranes of High Five insect cells overexpressing ABCG2 with different concentrations of A-803467. A-803467 stimulated the ATPase activity of ABCG2 in a concentration dependent manner, with a maximal stimulation of 2.13-fold greater than the basal activity (Fig. 3A). The inset of Fig. 3A reveals the concentration of A-803467 required to obtain 50% stimulation is 0.718 μM. Similarly, we assessed the effect of ABCG2 known substrates, topotecan and MX, on the ATPase activity of ABCG2. We measured ABCG2-mediated ATP hydrolysis in the presence of topotecan and MX at various concentrations from 0 to 10 μM. Interestingly, topotecan and MX stimulated the ATPase activity of ABCG2 in a concentration dependent manner, with a maximal stimulation of 1.81-fold and 2.04-fold greater than the basal activity, respectively (Fig. 3B and 3C). In addition, the concentration of topotecan and MX required to obtain 50% stimulation were 2.60 μM and 2.20 μM, respectively. These results suggest A-803467 interacts at the drug-substrate-binding site and stimulates the ATPase activity of ABCG2.

### Docking analysis of A-803467 with human ABCG2 homology model

**Figure 4:**
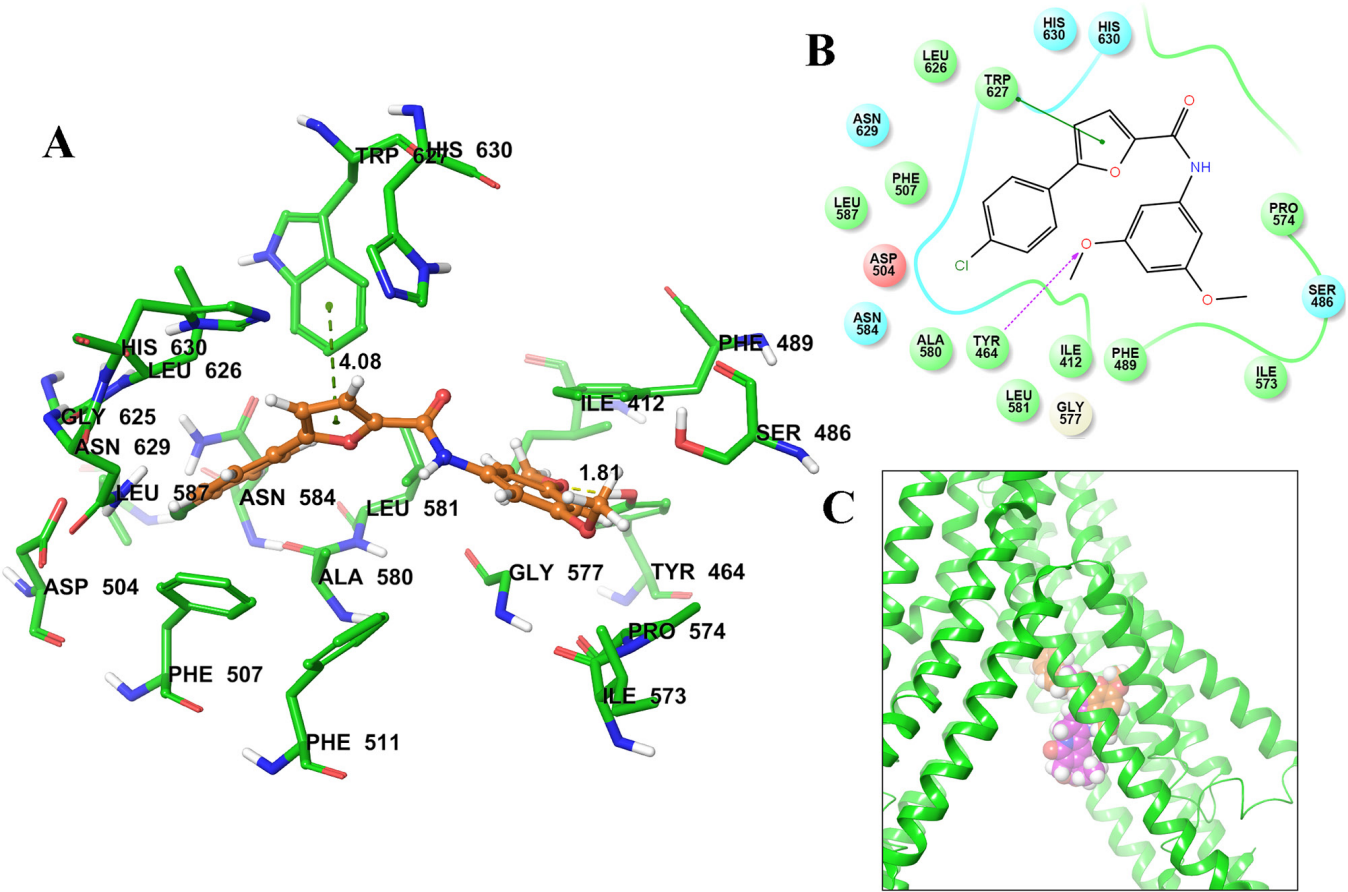
Predicted binding position of A-803467 with homology modeled ABCG2 **A.** Docked position of A-803467 within the drug-binding site of human ABCG2. Important residues are depicted as sticks with the atoms colored: carbon – green, hydrogen – white, nitrogen – blue, oxygen – red, sulfur – yellow. A-803467 is shown as ball and stick model with the same color scheme as above, except carbon atoms are represented in orange and chlorine in dark green. Ring centroids were represented as dark-green dots. Dotted yellow lines indicate hydrogen bonds. **B.** A two-dimensional ligand − receptor interaction diagram with important interactions observed in the docked complex of A-803467 with the drug-binding site residues of human ABCG2 is shown. The amino acids within 4 Å are shown as colored bubbles, red indicates negative charge, cyan indicates polar, and green indicates hydrophobic residues. Hydrogen bonds are shown by the purple dotted arrow, while π-π stacking aromatic interactions are shown by green lines. **C.** Location of predicted binding position of A-803467 (carbon atoms are represented in orange) and topotecan (carbon atoms are represented in purple) within transmembrane domain of ABCG2.

The best-scored docked position of A-803467 within the large drug-binding cavity of human ABCG2 is shown in Fig 4. The 4-chlorophenyl and furan carboxamide substituents of A-803467 were stabilized into a hydrophobic pocket formed by nearby residues Phe507, Ala580, Leu581, Asn583, Gly625, Leu626, Trp627, Asn629 and His630. The 4-chlorophenyl ring of A-803467 may be involved in a π-π interaction with the phenyl ring of Phe507 (centroid distance, 4.08 Å). The 3, 5-dimethoxyphenyl group of A-803467 was stabilized into the large cavity formed by side chains of hydrophobic residues Ile412, Tyr464, Ser486, Phe489, Ile573, Pro574 and Gly577. Moreover, the 3-methoxyphenyl group also entered into a hydrogen bonding interaction with Tyr464 (−O•••HO-Tyr464, 1.81 Å).

### A-803467 potentiates the anticancer activity of topotecan in ABCG2-overexpressing tumor xenograft model

**Figure 5:**
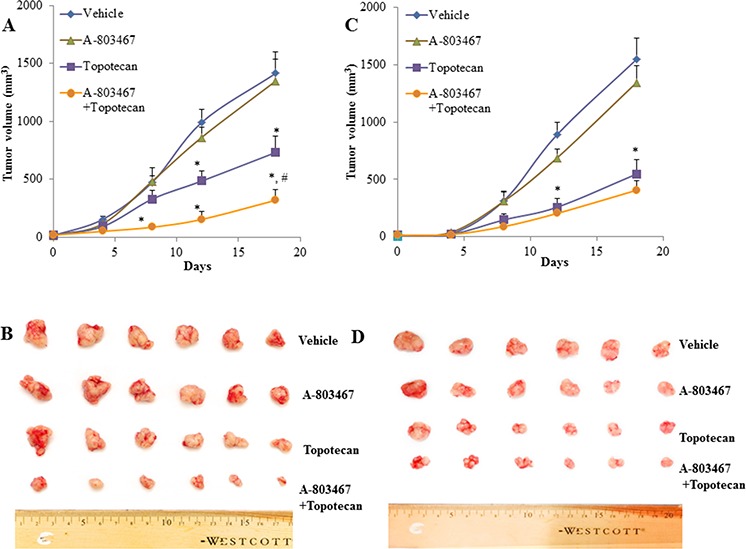
The effect of A-803467 on H460 and H460/MX20 tumor xenograft growth rate **A.** Changes in tumor volume with time in H460/MX20 xenograft are shown. Each point on line graph represents the mean tumor volume (mm^3^) at each particular day after implantation. **B.** A representative picture of the excised H460/MX20 tumors from the different mice on the 18th day after implantation. **C.** Changes in tumor volume with time in H460 xenograft are shown. **D.** A representative picture of the excised H460 tumors sized from different mice is shown on the 18th day after implantation. Each column represents the mean determinations and the bars represent SEM of 6 mice. *: *P* < 0.05 versus the vehicle group. ^#^: *P* < 0.05 versus the topotecan group.

**Figure 6:**
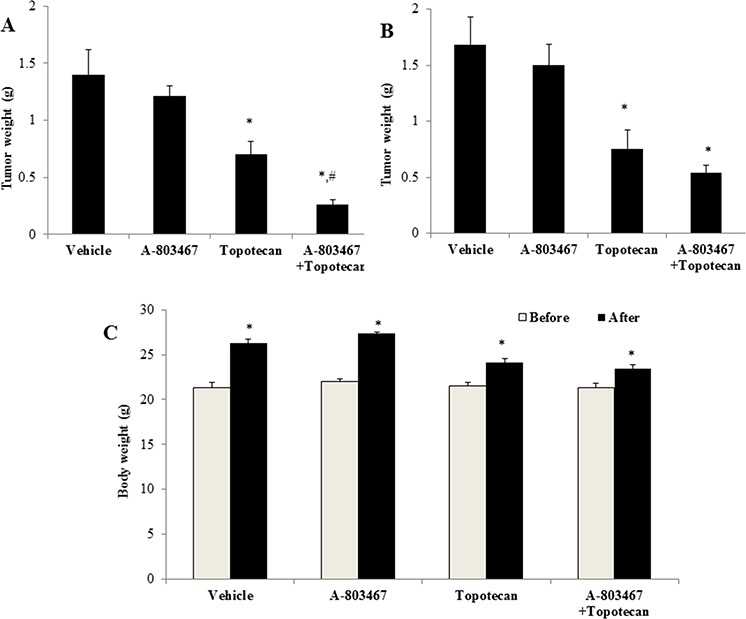
The effect of A-803467 on H460 and H460/MX20 tumor weight **A.** Mean H460/MX20 tumor weight (*n* = 6). **B.** Mean H460 tumor weight (*n* = 6). **C.** Changes in mean body weight before and after treatment for xenograft model. *: *P* < 0.05 versus the vehicle group; ^#^: *P* < 0.05 versus the topotecan group.

Parental H460 cells and drug resistant ABCG2 overexpressing H460/MX20 cells were implanted into athymic nude mice to create xenograft tumor models to analyze the efficacy of A-803467 to circumvent resistance to topotecan *in vivo*. A 35 mg/kg oral dose of A-803467 was chosen based on our preliminary study (data not shown) and showed no noticeable toxicity in the male NCR nude mice. Topotecan alone at 3 mg/kg i.p. dose demonstrated significant growth retardation in the parental H460 xenografts as well as ABCG2 overexpressing H460/MX20 xenografts (Fig. 5A–5D). However, the H460 tumor xenografts exhibited a more dramatic reduction when compared to H460/MX20 xenografts due to lack of ABCG2 expression in H460 tumors results in increased concentration of topotecan when compared to H460/MX20 tumors. The tumor growth rate of the xenograft mice implanted with ABCG2 overexpressing cells was significantly reduced in the A-803467-topotecan combination group as compared to the vehicle, A-803467 alone, and topotecan alone groups (Fig. 5A). Not only was the H460/MX20 tumor growth minimized, but also the size and weight of the tumors were significantly reduced in the combination treatment group (Fig. 5B and 6A). It should be mentioned that A-803467 alone had no significant effect on the growth rate of H460 (Fig. 5C and 6B) and H460/MX20 (Fig. 5A and Fig. 6A) xenografts. Furthermore, topotecan with or without A-803467 did not produce any apparent toxicity or weight loss (Fig. 6C). Overall, A-803467 did not present any increased toxicity in the mice, yet improved the efficacy of topotecan in the ABCG2 overexpressing H460/MX20 resistant xenograft model.

## DISCUSSION

ABCG2 plays an important role in development of drug resistance in clinical medicine. There is a strong correlation between ABCG2 overexpression and development of drug resistance in several cancer cells, including NSCLC, colon carcinoma, hepatocellular carcinoma (HCC) and breast cancer [37]. In the past four years, there has been rising evidence for overexpression of ABCG2 in hematologic malignancies and solid tumors; in these studies, ABCG2 overexpression significantly correlated with decreased patient survival [38]. The ABCG2 transporter is present in certain populations of cancer stem cells and normal primitive stem cells, increasing the likelihood of overexpression and thus resistance to various anticancer drugs [39–41]. Sensitizing these cells to anticancer drugs with the help of ABC transport reversal agents can effectively eradicate the tumor population, leading to better clinical outcomes for patients.

A wide variety of compounds that can inhibit ABCG2 have been comprehensively studied [42]. ABCG2 inhibitors include, fumitremorgin C (FTC), the FTC analogue Ko143, the acridone carboxamide derivative GF120918, anti-HIV protease inhibitors nelfinavir and ritonavir, the dietary flavonoids chrysin and biochanin A, the tyrosine kinase inhibitors gefitinib and imatinib [43], and some herbal extracts [44]. ABCG2 inhibitors such as Ko143, GF120918 and gefitinib are highly potent with their IC_50_ values in nano molar range. FTC and Ko143 are highly selective in inhibiting the ABCG2 transporter, whereas rest of the compounds seems to be general inhibitors of ABC transporters [42]. The selective ABCG2 inhibitors such as FTC and Ko 143 are effective only *in vitro* [45]. Several ABCG2 inhibitors have been identified but none of them are in clinical use due to toxicity and pharmacokinetic uncertainty. Hence there is still an ongoing search for a safer and specific inhibitor of the ABCG2 transporter.

In order to identify novel inhibitors of ABCG2, a cell based assay using MTT in ABCG2 overexpressing H460/MX20 cells was used to screen libraries of compounds. Similar approaches have been carried out by many researchers to obtain the inhibitors specific to ABCG2 [46]. In our study, we screened several tyrosine kinase inhibitors, synthetic small molecule inhibitors[47, 51] and ion channel inhibitors for activity in ABCG2 overexpressed cells and found that A-803467 was effective in inhibiting ABCG2 mediated drug resistance at micro molar concentrations.

One of the major findings of this study was that A-803467 significantly increased the sensitivity of ABCG2 overexpressing H460/MX20 cells to ABCG2 substrates such as topotecan and MX (Table 2). In addition, A-803467 did not enhance the cytotoxic effect of cisplatin, a drug that is not a substrate for the ABCG2 transporter, further demonstrating the specificity of A-803467. Moreover, previous studies have found that the 482^nd^ position in ABCG2 is a hot spot for mutation, Arg482 to Gly482 or Thr482 mutant variants of ABCG2 have shown to be significant in substrate specificity as well as the potency of ABCG2 antagonist [26, 52]. Robey et al. further reported that the activity of the ABCG2 transporter varies in these mutant cell lines. For example, novobiocin only antagonizes wild-type ABCG2 but does not show any effect in mutant variants. However, FTC has been shown to inhibit both wild-type as well as mutant ABCG2 [26]. This study has revealed that similar to FTC, A-803467 significantly enhances the chemosensitivity of ABCG2 substrates in both the cells with wild-type Arg482 and mutant Gly482 or Thr482 of ABCG2 (Table 1). These results clearly demonstrate the A-803467 activity in the aforementioned mutants of ABCG2. Furthermore, A-803467 could not reverse ABCB1- and ABCC10-mediated drug resistance in cells overexpressing ABCB1 and ABCC10 transporters (Table 3); thus implying that the reversal effect of A-803467 is ABCG2 specific.

In order to find the possible mechanism of action of A-803467, we investigated its effect on the ABCG2 expression. In this study, A-803467 at 7.5 μM did not significantly alter the expression of the ABCG2 protein in H460/MX20 cells (Fig. 2A). In addition, A-803467 did not translocate the ABCG2 protein from the cell membrane after 72 h of treatment (Fig. 2B). This clearly demonstrates that reversal of MDR by A-803467 is unlikely due to its decreasing ABCG2 protein expression or a translocation and most likely due to the interaction with ABCG2. Further functional analysis was performed by measuring the intracellular accumulation of [^3^H]-MX in wild-type HEK293/R482, mutant HEK293/R482T, and mutant HEK293/R482G cells (Fig. 1A). In addition, we also investigated the effect of A-803467 on the efflux of [^3^H]-MX in wild-type HEK293/R482 cells (Fig. 1B). A-803467 at 7.5 μM produced a significant increase in accumulation of MX by inhibiting the efflux function in the forenamed cell lines but not in parental HEK293/pcDNA3.1 cells.

To further understand the interaction of A-803467 with ABCG2, we performed an ATPase assay using ABCG2 overexpressed membranes. The majority of TKIs that interact with the ABC drug transporters stimulate ATP hydrolysis [53] and the fact that A-803467 stimulates the ATP hydrolysis of ABCG2 in a concentration dependent manner (Fig. 3A) indicates that it behaves similar to other known substrates (Fig. 3B and 3C) of ABCG2 transporter, such as MX and topotecan. These results further prove that A-803467 not only interacts directly with the ABCG2 transporter, but may also be a competitive inhibitor of the transporter.

To identify the molecular interaction of A-803467 with the ABCG2 transporter, docking simulation was performed at various sites of the human ABCG2 homology model. The crystal structure of human ABCG2 transporter is not completely elucidated. Comparing the docking scores shown in Table 4, the most favorable binding site was identified as site-1. Molecular docking of topotecan, a well-known ABCG2 substrate, at the same site of ABCG2 was performed. The docking score of topotecan (−5.57 kcal/mol) is much higher than that of A-803467 (−8.07 kcal/mol). The lower docking score indicates stronger interaction between A-803467 to ABCG2 (Fig. 4). Moreover, molecular structure of A-803467 also exhibited the pharmacophoric features such as hydrophobic groups, aromatic ring centers (phenyl ring and furan ring) and hydrogen bond acceptors that have been reported as essential for ABCG2 inhibition [54]. Overall, this molecular simulation will provide clues to optimize further derivatives of ABCG2 inhibitors.

**Table 4:** Glide docking scores of A-803467 and topotecan within in each of the predicted binding sites of ABCG2

Binding sites	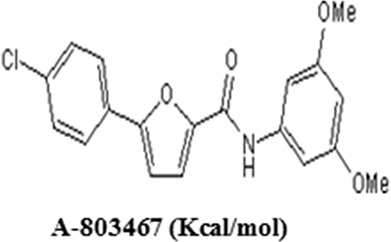	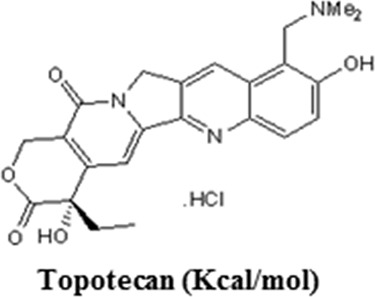
**Site-1^a^**	–8.07	–5.57
**Site-2^b^**	–6.78	NA
**Site-3^c^**	–3.89	NA
**Site-4^d^**	–2.83	NA

Binding energies of A-803467 and topotecan within each of the predicted binding sites of ABCG2.

a Site grid generated using Arg482:

b Site grid generated using Asn629:

c Site grid generated using Arg383:

d Site grid generated using Leu241 and Gly832.

To our knowledge, this is the first study that demonstrates the combined effect of A-803467 with anticancer drug topotecan in NCR nude mice implanted with ABCG2 overexpressing H460/MX20 cells. The dose and route of administration of A-803467 (35 mg/kg, p.o) was chosen based on our preliminary study (data not shown). A-803467 was administered on the 2^nd^ and 3^rd^ day before administration of topotecan to compensate for its high binding to plasma proteins. Jarvis et al., reported that A-803467 was highly bound (98.7%) to plasma proteins in rats [20]. The *in vivo* study results indicated that A-803467 in combination with topotecan, significantly decreased the tumor growth in mice implanted with ABCG2 overexpressing H460/MX20 cells (Fig 5A, 5B and 6A). A-803467 effectively restored the sensitivity of tumors overexpressing ABCG2 transporter to topotecan without having any significant effect on tumors lacking ABCG2 expression (Fig. 5C, 5D and 6B). Furthermore, A-803467 alone, or in combination with topotecan, did not produce significant observable toxicity or weight loss during the study period (Fig. 6C). The safety profile of A-803467 in humans is not investigated yet, but currently A-803467 is in pre-clinical testing stage for neuropathic and inflammatory pain [20]

ABCG2 is responsible for the “side population” (SP) phenotype, frequently used in the identification and isolation of cancer stem cells (CSCs). ABCG2 has also been suggested as a prognostic biomarker as well as a novel therapeutic target for the eradication of CSCs [40]. Presently, conventional chemotherapeutic anticancer agents target highly proliferative tumor cells. The CSCs survive such chemotherapy due to their high expression of ABCG2 transporter, mediating their chemoresistance, and ultimately leading to tumor relapse and metastasis. Completely eradicating cancer stem cells by overcoming the resistance to chemotherapy, mediated by ABCG2, would be a new targeted therapeutic strategy. This study suggests that combined therapy of A-803467 with ABCG2 substrates may provide a more effective way of sensitizing ABCG2-mediated MDR and possibly eliminating CSCsIn conclusion, A-803467, a tetrodotoxin resistant sodium channel blocker, effectively inhibits membrane ABCG2 function, without affecting its expression or cellular location and re-sensitizes the ABCG2 substrates in ABCG2 overexpressing cells. A-803467, even at micro molar concentrations, stimulates ATP hydrolysis of the ABCG2 transporter with strong binding interactions at the transmembrane site of the transporter. A-803467 significantly potentiates the antitumor efficacy of topotecan in tumor xenograft nude mice. Therefore, it is likely that A-803467, in combination with anticancer agents that are ABCG2 substrates, would be very useful in the treatment of certain drug resistant cancers.

## MATERIALS AND METHODS

### Chemicals

A-803467 was purchased from Alomone Labs (Jerusalem, Israel). MX, topotecan, vincristine, cisplatin, verapamil, dimethyl sulfoxide (DMSO), ammonium molybdate, MES hydrate, antimony potassium tartrate, sodium azide, N-methyl-D-glucamine and 3-(4,5-dimethylthiazole-2-yl)-2,5-biphenyltetrazolium bromide (MTT) were purchased from Sigma Chemical Co (St. Louis, USA). [^3^H]-MX (4 Ci/mmol) was purchased from Moravek Biochemicals, Inc (Brea, CA). Dulbecco's modified Eagle's medium (DMEM), fetal bovine serum (FBS), penicillin/streptomycin and trypsin 0.25% were purchased from Hyclone (Waltham, MA). Monoclonal antibodies BXP-21 (against ABCG2), sc-8432 (against actin) and horseradish peroxidase-labeled anti-mouse IgG were purchased from Santa Cruz Biotechnology, Inc. (Santa Cruz, CA). Alexa flour 488-conjugated goat anti-mouse IgG was purchased from Molecular Probes (Eugene, OR). Full-Range Rainbow Molecular weight marker was purchased from GE Healthcare Life Sciences (Pittsburgh, PA). Potassium phosphate, EGTA and ATP were purchased from AMRESCO (Solon, OH). Sulfuric acid solution (37 N) was purchased from Fisher Scientific (Pittsburgh, PA). KCl was purchased from Avantor Performance Materials (Center Valley, PA). Ouabain was purchased from Enzo Life Sciences, Inc. (Farmingdale, NY). Dithiothreitol was purchased from Promega Corporation (Madison, WI). MgCl_2_ was purchased from EMD Millipore (Billerica, MA). Ascorbic acid was purchased from VWR International (West Chester, PA). Sodium orthovanadate was purchased from Alfa Aesar (Ward Hill, MA). Cepharanthine was provided by Kakenshoyaku Co. (Tokyo, Japan). Fumitremorgin C (FTC) was provided by Dr. Susan E. Bates (Bethesda, USA).

### Equipment

OPSYS microplate reader was purchased from Dynex Technologies (Chantilly, VA). Packard TRI-CARB1 1900CA liquid scintillation analyzer was purchased from Packard Instrument Company, Inc (Downers Grove, IL). Nikon eclipse TE2000-S fluorescence microscope was purchased from Nikon (Melville, NY).

### Cell lines and cell culture

HEK293/pcDNA3.1, wild-type HEK293/R482, mutant HEK293/R482T and mutant HEK293/R482G cells were established by transfecting HEK293 cell with either the empty pcDNA3.1 vector or pcDNA3.1 vector containing a full-length ABCG2, with coding arginine (R), threonine (T), or glycine (G) at amino acid position 482, respectively, after selection with G418 and maintained in medium with 2 mg/ml of G418 [26]. HEK293/ABCB1 and HEK293/ABCC10 cell lines were generated by selection with G418 (2 mg/ml) after transfecting HEK293 cell with ABCB1 vector or ABCC10 vector, respectively [27]. The human lung cancer cell line H460, and its MX-selected derivative ABCG2-overexpressing cell line H460/MX20 were used in the study [28]. All cell lines were maintained in RPMI 1640 or DMEM medium, containing 10% fetal bovine serum and 1% penicillin/streptomycin and cultured in an incubator at 37°C with 5% CO_2_.

### Cell viability assay

Cytotoxicity tests and reversal experiments were performed using the MTT colorimetric assay as described previously [29]. Cells were harvested and resuspended in a final concentration of 6 × 10^3^ cells/well for HEK293/pcDNA3.1, HEK/ABCB1, HEK/ABCC10, HEK293/R482, HEK293/R482G and HEK293/R482T cells, and 4 × 10^3^ cells/well for H460 and H460/MX20 cells. Cells were seeded evenly into 96-well plates. To determine the cytotoxicity of A-803467, different concentrations of drug were added into the each well after 24 h of incubation. To determine the reversal capability of A-803467, various concentrations of chemotherapeutic drugs were added into designated wells after 2 h preincubation with A-803467, FTC, verapamil or cepharanthine. After 68 h of drug incubation, MTT reagent (4 mg/mL) was added. The plates were incubated for an additional 4 h, the supernatant was discarded and 100 μl of DMSO were added to dissolve the formazan crystals. Cell viability was measured at a wavelength of 570 nm. All the experiments were repeated at least 3 times, and the mean and standard deviation (SD) values were calculated.

### [^3^H]-MX accumulation and efflux assay

We examined the effect of A-803467 on the intracellular accumulation and efflux of [^3^H]-MX in ABCG2-overexpressing cells as previous described [30]. Briefly, the cells (5 × 10^6^/cells) were resuspended and incubated in the RPMI 1640 medium in the presence or absence of A-803467 (7.5 μM) or FTC (5 μM) at 37°C for 2 h. Cells were then incubated with 0.01 μM [^3^H]-MX containing medium for additional 2 h at 37°C, with or without A-803467 (7.5 μM) or FTC (5 μM), and subsequently washed twice with ice-cold PBS. For the accumulation assay, cells were lysed by the 10 mM lysis buffer (pH 7.4, containing 1% Triton X-100 and 0.2% SDS) and then placed in scintillation fluid. For the efflux assay, the suspended cells were then cultured in [^3^H]-MX free medium, with or without A-803467 (7.5 μM) or FTC (5.0 μM) at 37°C. The aliquots of cells were harvested at the indicated times (0, 30, 60, and 120 min), and then washed with ice-cold PBS and transferred to respective scintillation vials. The radioactivity was measured using the Packard TRI-CARB1 190`A liquid scintillation analyzer.

### Western blot analysis

Cell lysates were prepared as described previously [31]. Equal amounts of total cell lysates (30 μg protein) were resolved by sodium dodecyl sulfate polyacrylamide gel electrophoresis (SDS-PAGE) and electrophoretically transferred onto polyvinylidene fluoride (PVDF) membranes. After incubation in a blocking solution (5% milk) for 1 h at room temperature, the membranes were immunoblotted overnight with primary monoclonal antibodies against actin at 1:1000 dilution or ABCG2 at 1:500 dilution at 4°C, and were then further incubated for 2 h at room temperature with horseradish peroxide (HRP)-conjugated secondary antibody (1:1000 dilution). The protein–antibody complex was detected by enhanced chemiluminescence detection system (Amersham, NJ).

### Immunofluorescence analysis

For immunofluorescence analysis, H460 and H460/MX20 cells were seeded in 24 well plates. Cells were incubated with or without A-803467 (7.5 μM) for 72 h. Thereafter, cells were washed with PBS and fixed with 4% paraformaldehyde for 15 min at room temperature and then rinsed with PBS three times, followed by permeabilization with 1% triton X-100 for 10 min at 4°C. Cells were again washed for three times with PBS, and then blocked with 2 mg/ml of BSA for 1 h at 37°C. Fixed cells were incubated with monoclonal antibody against ABCG2 (BXP 21) (1:50) for 16 h at 4°C, followed by three washes with PBS. The cells were then further incubated with Alexa flour 488 goat anti-mouse IgG (1:60) for 1 h at 37°C. DAPI was used for nuclear counterstaining. Immunofluorescence images were taken with a Nikon fluorescence microscope.

### ABCG2 ATPase assay

The Vi-sensitive ATPase activity of ABCG2 in the membrane vesicles of High Five insect cells was measured as previously described. The membrane vesicles (100 μg protein/ml) were incubated in ATPase assay buffer with or without 0.3 mM vanadate at 37°C for 5 min and then incubated with different concentrations of A-803467 ranging from 0 to 80 μM, topotecan, and MX (0 – 30 μM), at 37°C for 3 min. The ATPase reaction was induced by the addition of 5 mM Mg-ATP, and the total volume was 0.1 mL. After incubation at 37°C for 20 min, the reactions were stopped by loading 0.1 mL of 5% SDS solution. The liberated inorganic phosphate (Pi) was measured as described previously [28]

### Molecular modeling

A-803467 was prepared as ligands for docking simulation onto human ABCG2 homology model following the same protocols as previously described [32]. All grids of ABCG2 were prepared and generated as per our previous protocols [32]. Grid-1 generated using residue Arg482 as the centroid had the highest docking score; therefore, docking discussion was based on binding position of A-803467 at this site. Glide v6.0 (Schrödinger, LLC, New York, NY, 2013) docking protocol was followed with the default parameters. Top scoring conformation was used for graphical analysis. All computations were carried out on a Dell 490n dual processor with Linux OS (Ubuntu 12.04 LTS).

### Animals

Athymic NCr (nu/nu) nude mice, weighing 18 to 22 g (Taconic Farms, NCRNU-M, Homozygous, Albino color), were used for the ABCG2 xenograft models. All animals were provided with sterilized water and rodent chow ad libitum and maintained with an alternating 12 h light/dark cycle. All the experiments were approved by the Institutional Animal Care & Use Committee (IACUC) of St. John's University, and were carried out in accordance with the guidelines from Animal Welfare Act and The U.S. Public Health Service.

### Nude mouse MDR xenograft models

The ABCG2-overexpressing NSCLC cell H460/MX20 xenograft mouse models were established as previously explained [41]. H460/MX20 cells (6 × 10^6^) and H460 cells (4 × 10^6^) were injected subcutaneously under the right and left armpit regions of the nude mice, respectively. We performed a pilot study using three different doses of A-803467 (17.5, 35 and 70 mg/kg) and we found that 35 mg/kg dose was effective in increasing the topotecan sensitivity in tumors without significantly increase toxicity, therefore 35 mg/kg dose was used throughout the following study.

The mice were randomized into 4 groups (*n* = 6) when the tumors attained a mean diameter of 0.5 cm (day 0), and then received treatments as follows: (a) Vehicle (10% N-methyl pyrrolidine (NMP) in PEG-300, p.o., every 2^nd^ and 3^rd^ day; total 12 times), (b) A-803467 diluted in 10% NMP in PEG-300 (35 mg/kg, p.o., every 2^nd^ and 3^rd^ day; total 12 times), (c) Topotecan (3.0 mg/kg, i.p., every 3^rd^ day; total 6 times), and (d) A-803467 (35 mg/kg, every 2^nd^ and 3^rd^ day; total 12 times, given 1 h before topotecan) + topotecan (3.0 mg/kg, i.p., every 3rd day: total 6 times). The body weights of the mice were monitored and the two perpendicular diameters of tumors (*A* and *B*) were recorded every 4^th^ day, and tumor volumes (*V*) were calculated according to the following formula described previously [41].

$$\text{V}=\frac{\pi }{6}{\left(\frac{A+B}{2}\right)}^{3}$$

The ratio of growth inhibition (IR) described previously [41] was estimated according to the formula given below.

$$\text{IR}(\%)=1-\frac{\text{Mean tumor weight of experimental group}}{\text{Mean tumor weight of control group}}\times 100$$

### Statistical analysis

All experiments were repeated at least three times and the differences were determined by using the one-way ANOVA followed by Newman-Keuls post hoc test for comparing multiple groups with one variable in the following experiments: cell viability assay, accumulation assay, quantification of immunoblotting and tumor weight measurement. Statistical analysis was performed by two way ANOVA followed by Bonferroni post hoc test for comparing multiple groups with more than one variable in the following experiments: efflux assay, tumor growth rate measurement and body weight measurement. Statistical analysis was performed by un-paired student *t*-test for comparing two groups in immunoblotting. Statistical significance was set at *P* < 0.05. Statistical analysis was performed using GraphPad Prism version 6.01 for Windows (GraphPad Software, La Jolla, CA).

## SUPPLEMENTARY FIGURES


