# Supplementary material for: A-803467, a tetrodotoxin-resistant sodium channel blocker, modulates ABCG2-mediated MDR in vitro and in vivo
Source: Oncotarget. 2015 Oct 22;6(36):39276–91. doi: 10.18632/oncotarget.5747 (PMC4770772; doi:10.18632/oncotarget.5747)
Supplement: Supplementary file 1 [file oncotarget-06-39276-s001.pdf]

SUPPLEMENTARY FIGURES

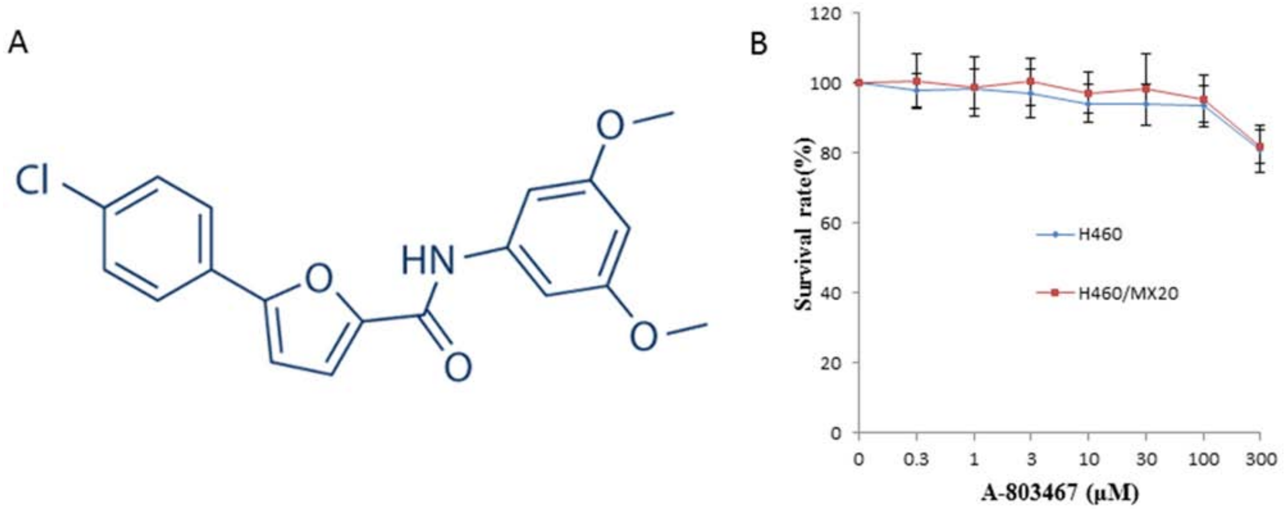

Supplementary Figure S1: The chemical structure of A-803467 and the effect of A-803467 on the cell lines used in the study. A. The chemical structure of A-803467 (5-(4-chlorophenyl)-N-(3,5-dimethoxyphenyl)furan-2-carboxamide). **B.** Cytotoxicity of A-803467 in H460 and H460/MX20 cell lines.

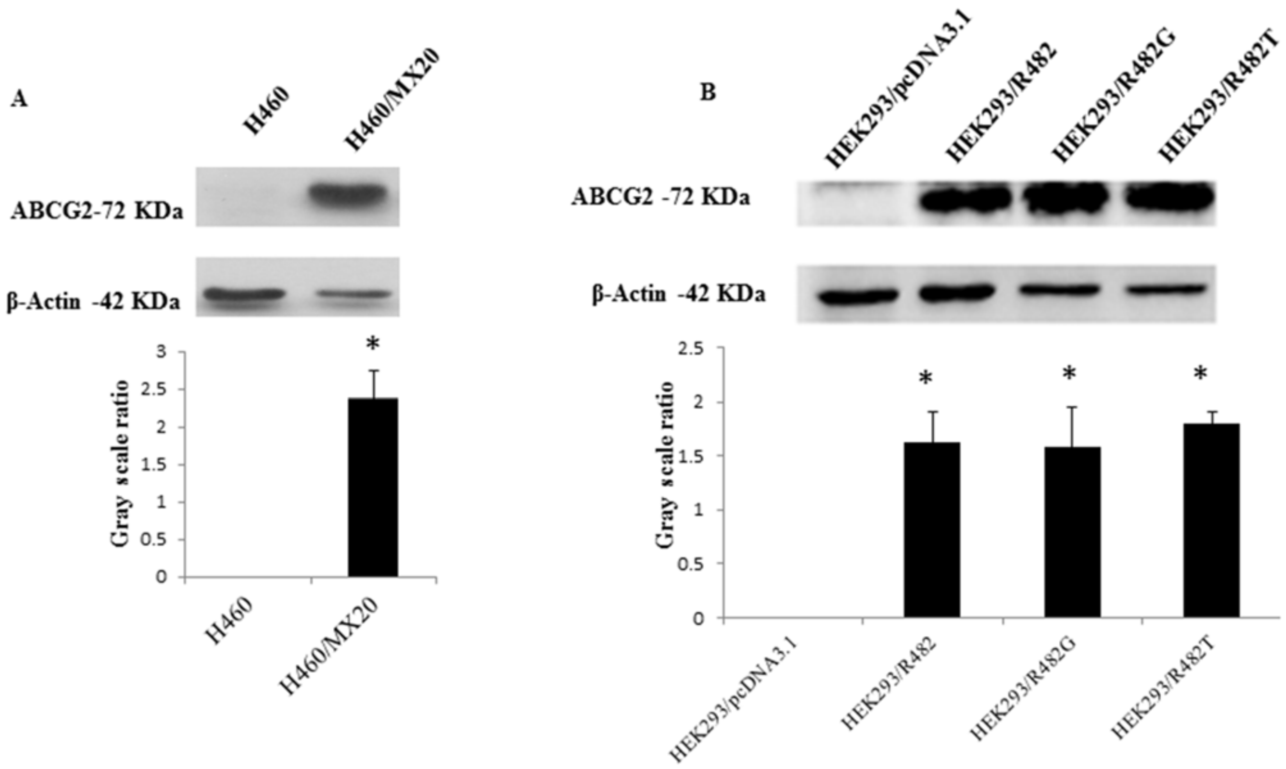

Supplementary Figure S2: Expression of ABCG2 transporter in cell lines used in the study. A. Western blotting to detect ABCG2-expression in H460 and in ABCG2-overexpressing H460/MX20 cell line **B.** Transfected HEK293/R482, HEK293/R482G, and HEK293/R482T cell lines. The protein levels of ABCG2 were normalized to those of β-actin. Representative results are shown here and similar results were obtained in other two trials. Values are the mean ± SD of 3 assays.
